# High Levels of Heavy Metal(loid)s Related to Biliary Hyperplasia in Hedgehogs (*Erinaceus europaeus*)

**DOI:** 10.3390/ani13081359

**Published:** 2023-04-15

**Authors:** Catarina Jota Baptista, Fernanda Seixas, José M. Gonzalo-Orden, Carla Patinha, Pedro Pato, Eduardo Ferreira da Silva, María Casero, Erica Brazio, Ricardo Brandão, Daniela Costa, Teresa Letra Mateus, Paula A. Oliveira

**Affiliations:** 1 Departamento de Ciências Veterinárias, Escola de Ciências Agrárias e Veterinárias (ECAV), Universidade de Trás-os-Montes e Alto Douro, UTAD, 5000-801 Vila Real, Portugal; 2 Centro de Investigação das Tecnologias Agroambientais e Biológicas (CITAB), UTAD, 5000-801 Vila Real, Portugal; 3 Instituto de Biomedicina (IBIOMED), Universidad de León, 24071 León, Spain; 4 Faculdade de Medicina Veterinária, Universidade Lusófona de Humanidades e Tecnologias, 1749-024 Lisboa, Portugal; 5 Centro de Ciência Animal e Veterinária (CECAV), AL4AnimalS, UTAD, 5000-801 Vila Real, Portugal; 6 GeoBioTec, Departamento de Geociências, Universidade de Aveiro, 3810-193 Aveiro, Portugal; 7 RIAS-ALDEIA—Wildlife Rehabilitation and Research Centre, Parque Natural da Ria Formosa, 8700-194 Olhão, Portugal; 8 Centro de Recuperação dos Animais Silvestres de Lisboa (LxCRAS), Parque Florestal de Monsanto, 1500-068 Lisboa, Portugal; 9 CERVAS-ALDEIA—Centro de Ecologia, Recuperação e Vigilância de Animais Selvagens, 6290-520 Gouveia, Portugal; 10 CISAS-Centre for Research and Development in Agrifood Systems and Sustainability, Escola Superior Agrária, Instituto Politécnico de Viana do Castelo, 4990-706 Viana do Castelo, Portugal; 11 EpiUnit—Instituto de Saúde Pública da Universidade do Porto, Laboratory for Integrative and Translational Research in Population Health, 4050-600 Porto, Portugal

**Keywords:** metal, biliary hyperplasia, hepatotoxicity, histopathology, wildlife, One Health

## Abstract

**Simple Summary:**

Heavy metal(loid)s are hazardous substances for humans, animals and ecosystems. The liver is one of the most affected organs, presenting lesions after being acutely or chronically exposed to these substances. In this study, hepatic metal(loid)s’ concentrations were associated with biliary hyperplasia, which was the most common hepatic lesion found in a group of western-European hedgehogs from rescue centres in Portugal. With exception of arsenic (As), all metal(loid)s were present in higher concentrations in animals with biliary hyperplasia. Further research is necessary to support these results and clarify the molecular mechanisms that lead to hepatic lesions provoked by these compounds.

**Abstract:**

Heavy metal(loid) pollution of ecosystems is a current One Health problem. The liver is one of the most affected organs in cases of acute or chronic exposure to abnormal amounts of these substances, inducing histopathologic lesions. In order to assess the influence of heavy metal(loids), forty-five European hedgehogs (*Erinaceus europaeus*) were submitted to necropsy, and liver samples were collected for a routine histopathology exam and metal(loid)s determination (As, Cd, Co, Cr, Cu and Pb) by ICP-MS. Age was estimated during the necropsy exam. Biliary hyperplasia was the most frequent lesion observed (16/45; 35.56%). No statistically significant associations were found between biliary hyperplasia and age or sex. Metal(loid)s’ concentrations were higher in animals with biliary hyperplasia (except for As). There was a statistically significant difference for both Cd and Co. For As, Cd and Co, cubs and juveniles animals showed significantly lower concentrations than elder individuals. Only for Pb were significant differences found between females and males. As described in the literature, exposure to metal(loid)s may be a cause of biliary hyperplasia, although further research (including the use of biochemical methods) is needed to support these results. To the authors’ knowledge, this is the first report of this association in hedgehogs.

## 1. Introduction

Heavy metal(loid) (e.g., arsenic, As; cadmium, Cd; cobalt, Co; chromium, Cr; copper, Cu; and lead, Pb) pollution is a current One Health global problem, affecting multiple ecosystems worldwide, and consequently animals and humans [1,2,3].

Inhalation, ingestion and dermal contact are the most relevant sources of exposure to heavy metal(loid)s for animals and humans [4]. Absorption of large amounts of these compounds may lead to acute intoxication and sudden death. On the other hand, their potential to be perpetuated in the environment, bioaccumulate in trophic chains and accumulate in certain organs, may lead to chronic lesions in different organisms and tissues. For instance, some can be hepatotoxic, nephrotoxic, neurotoxic, genotoxic and even carcinogenic. Both essential (such as Cr or Cu, which participate in some biological processes) and non-essential (such as Cd or Pb, which have no biological function) metal(loid)s can be toxic, depending on the dose [2]. Aside from their hazardous effects on animals’ health, these metal(loid)s clearly interfere with the nutritional value of plants (namely, carbohydrates, proteins and vitamins). As a consequence, the consumption of contaminated plants affects food webs, habitats and, in the end, terrestrial ecosystems.

Even though some of these consequences may be shared by many specimens, some animals, including western-European hedgehogs (*Erinaceus europaeus*), are considered particularly suitable to evaluating this health hazard, due to biological and ecological aspects (e.g., food regimen, space distribution, resilience and habits) [5,6,7,8]. *E. europaeus* is cosmopolitan, largely distributed (in natural, rural and urban areas) and has a mainly insectivorous diet which includes large amounts of lipids, and these usually accumulate metal(loid)s [5,8]. The use of sentinels allows researchers to have a more complete and anticipated perspective of any hazard and allows on-time implementations of the necessary mitigation strategies [9,10,11].

The liver is one of the most used organs for biomonitoring and studying the effects of heavy metal(loid)s, mainly due to its role in several metabolic and detoxification processes [12,13]. The hepatoxicity induced by heavy metal(loid)s is a complex of biochemical and cellular alterations. In abnormally high concentrations, metal(loid)s are responsible for oxidative stress reactions, which mean imbalances in the production of radicals and oxidant compounds, and their elimination by antioxidants [14]. Depending on the metal(loid) or the availability of other compounds (e.g., metallothioneins and selenium) that may potentiate or suppress some chemical reactions, their real effects on liver tissue may variate [14]. Although the hepatoxicity of Pb is considered the most well-known among all metal(loid)s, its exact mechanism of its toxicity is still not completely understood [12]. Nuclear erythroid 2-related factor (Nrf2) has been proposed to be intimately involved in heavy metal hepatotoxicity, and therefore, it has been used as a target to discover hepatoprotective agents [14]. In general, hepatotoxicity by heavy metal(loids) may be detected elevated levels of liver enzymes (e.g., aspartate aminotransferase, AST; alanine aminotransferase, ALT; or alkaline phosphatase, ALP); elevated levels of necrotic and transforming growth factors; and histopathologic changes. Histopathologic liver changes due to metal(loid) toxicity include (but are not limited to) hydropic alterations, oedema, congestion, inflammatory cell infiltration, necrosis, fibrosis, steatosis and biliary hyperplasia [14,15,16,17]. Histopathology plays an essential role as a biomarker of the effect of metal(loid)s’ toxicosis, when associated with the detection and measurement of these compounds in biological samples [18,19].

The aim of this study was to relate and interpret the concentrations of some metal(loid)s with health importance in relation to an important histopathology finding (biliary hyperplasia) in a sentinel species (*E. europaeus*).

## 2. Materials and Methods

### 2.1. Necropsies and Liver Samples’ Collection

All the hedgehogs in this study died or were found dead and delivered to one of three Portuguese rescue centres (CERVAS, LxCRAS and RIAS—in the north (n = 15), centre (n = 12) and south (n = 19), respectively). CERVAS is located in Gouveia, Guarda, Portugal; LxCRAS is Located in Parque Florestal de Monsanto, Lisbon, Portugal; and RIAS is located in Parque Natural da Ria Formosa, Olhão, Portugal. Hedgehogs in this study originally from eight districts of Portugal were sampled: Viana do Castelo (n = 1), Viseu (n = 5), Guarda (n = 4), Coimbra (n = 4), Castelo Branco (n = 1), Great Lisbon (n = 10), Setúbal (n = 2) and Faro (n = 18). None of them was killed for the study; they all died naturally or were euthanized according to the rescue centre’s internal policy, between 2019 and 2021. A complete macroscopic exam was performed for forty-five hedgehogs. Sex and age group (cub or pre-weaned, juveniles or post-weaned and adult) were estimated according to methods described in the literature [20]. A small portion (1.5 cm × 1.5 cm) of the liver was collected and kept in 10% buffered formaline for histopathological analysis. Approximately 6 g of the liver was collected in a zip bag and stored under −10 °C until chemical analysis.

### 2.2. Histopathology

Liver samples were analysed in the Histopathology Laboratory at the University of Trás-os-Montes and Alto Douro (UTAD). They were cut into small slices (2–3 mm) and processed for light microscopy according to routine technique. A blinded histopathologic evaluation was performed using an optical microscope (Nikon E600^®^, Nikon Instruments Inc., Melville, NY, USA).

### 2.3. Metal(loid)s Determination

Twenty-four hours before lyophilisation, forty-two liver-frozen samples were transferred to a −20 °C freezer. Then, samples were completely freeze-dried for 48 h at −56 °C (LaboGene CoolSafe^®^, Allerød, Denmark). Weight was registered before and after lyophilisation (Kern ALT^®^ Germany precision scale) in order to calculate the percentage of humidity of the tissue removed during the lyophilisation process (average value of 75.1%).

Approximately 0.5 g of each dried liver was weighed on the precision scale and transferred to digestion tubes. Then, 1 mL of nitric acid was added to each tube, which was left at room temperature overnight. Then, 2 mL of hydrogen peroxide was added to each sample. After 5 h at room temperature, samples were placed on a digestion plate (DigiPrep-MS^®^, Canada), where the temperature increased progressively for 15 min until reaching 85 °C. Then, it remained at 85 degrees for another 15 min. All the samples were adequately digested after this cycle and presented no visible solid particles. The concentrations of arsenic (As), cadmium (Cd, chromium (Cr), cobalt (Co), copper (Cu) and lead (Pb) were determined by an Agilent 7700 inductively coupled plasma mass spectrophotometer (ICP-MS) (Agilent Technologies^®^, Santa Clara, CA, USA). Certified reference material (ERM BB185^®^, Belgium), and blank tubes and duplicates were used for quality control of the mentioned procedures. Results were accepted when recoveries ranged between 70% and 120%. Average quantification limits (AQL) were 0.0125 mg/kg for As, 0.005 mg/kg for Cd, 0.0025 for Co, 0.005 mg/kg for Cr, 0.005 mg/kg for Cu and 0.005 mg/kg for Pb. Thus, values below these limits were assumed as zero.

### 2.4. Statistical Analysis

IBM SPSS^®^ Statistics 27 was used for descriptive analysis and statistical tests. Normality tests were applied to the quantitative data (Kolmogorov–Smirnov and Shapiro–Wilk tests), revealing a non-normal distribution. Then, data were log-transformed (Log10(x)) to get normal distributions [21]. Levene’s test was performed to verify that there was homogeneity of variances. Analysis of variance (One-way ANOVA) was then applied to all the samples according to the age group, sex and presence of biliary hyperplasia. Tukey’s HSD test was performed as a post hoc test for ANOVA when more than two groups were analysed. Chi-square tests were performed to evaluate statistical associations among sex, age and the presence of biliary hyperplasia. A binary logistic regression model was used to analyse the influences of age, sex and the metal(loid)s’ concentrations (all together) on the presence of biliary hyperplasia. For all the statistical tests, a critical *p*-value of 0.05 was considered.

## 3. Results

### 3.1. Histopathology

**Figure 1:**
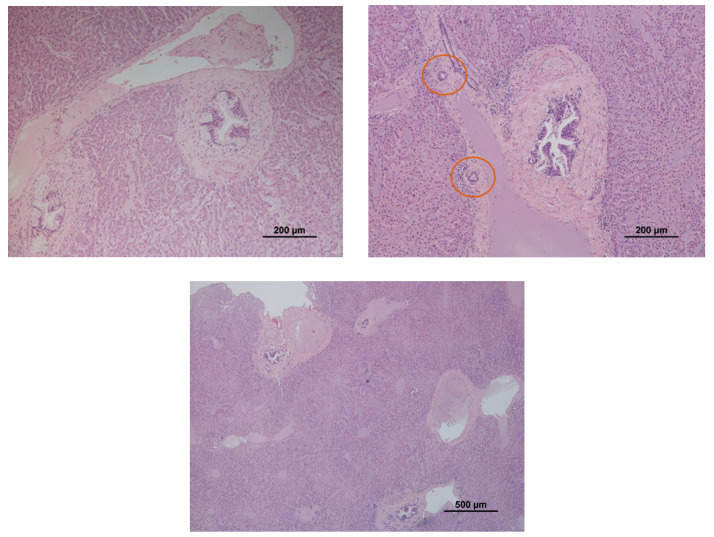
Evidence of biliary hyperplasia detected in a hedgehog’s liver (evident enlarged duct walls and lumen). Orange circles indicate some normal biliary ducts for comparison. Note that the hedgehog carcasses were frozen before the necropsy, so it is possible to notice some freezing artefacts.

A total of 45 hedgehogs’ livers were analysed for histopathology. Most livers were in autolysis (28/45; 62.22%). Biliary hyperplasia was the most diagnosed alteration observed (16/45; 35.56%) (Figure 1). Other changes were detected with lower prevalence, such as the presence of inflammatory infiltrates (7/45; 15.56%), steatosis (3/45; 6.67%) and subacute hepatitis (2/45; 4.44%). No statistically significant associations were found between biliary hyperplasia and age (F = 3.515; df = 2; *p* = 0.173) or sex (F = 3.019; df = 1; *p* = 0.082).

### 3.2. Metal(loid)s Determination

The mean, standard deviation (SD), minimum (Min.) and maximum (Max.) for each metal are presented in Table 1. Descriptive statistics are presented separately for animals with and without biliary hyperplasia (BH). The higher values were found for Cu (35.66 ± 19.89 mg/kg dry weight (dw). Maximum values surpassed 100 mg/kg dw. Cr was the scarcest metal (0.12 ± 0.11 mg/kg dw). With the exception of As, all metals had higher mean values in hedgehogs with BH than in animals without; and these differences were statistically significant for Cd and Co (*p* = 0.007 and *p* = 0.019, respectively) (Table 2).

Moreover, the mean values for all metals were higher in adults (n = 22) compared to cubs (n = 11) and juveniles (n = 9). These differences were statistically significant for As (F = 5.082; df = 2; *p* = 0.012), Co (F = 6.301; df = 2; *p* = 0.004) and Cd (F = 27.656; df = 2; *p* < 0.001). According to the Tukey HSD test, for As, juveniles’ levels were significantly lower than those of adults and cubs; and for Cd and Co, both cubs and juveniles had significantly lower levels than adults. No statistically significant differences were found between females (n = 23) and males (n = 19), except for Pb (F = 5.054; df = 1; *p* = 0.030).

The binary logistic regression model showed a high Hosmer and Lemeshow probability result of 0.993, and moderate accuracy of 75.7%. None of the variables was statistically significant. However, Log10(Co) and Log10(Pb) resulted in the highest Exp(B) values of 16.392 and 9.248, respectively (Appendix A).

Regarding the three rescue centres (CERVAS; LxCRAS and RIAS), no statistically significant differences were found between them for the presence of BH (F = 4.068; df = 2; *p*= 0.131).

## 4. Discussion

Livers from *E. europaeus* were collected and analysed for histopathology and metal determination (As, Cd, Co, Cr, Cu and Pb). Correlations with sex, age group and the presence of biliary hyperplasia were calculated. Metal(loid)s’ results from this study were separately interpreted and compared to those of other hedgehog and small-mammal studies (other authors’ results were converted to mg/kg when presented in other units for easier comparison).

As is a highly carcinogenic metalloid in its inorganic form. Chronic exposure to arsenicals by inhalation, ingestion or other routes has been related to liver disease and other conditions. In mammals, As poisoning is commonly manifested by acute signs, but chronic poisoning is rarely seen; and its metabolism and effects are severely affected by the species and the chemical form of As used [22]. The present study revealed small concentrations of As in the liver (0.13 ± 0.14 mg/kg dw) in animals with and without biliary hyperplasia when compared with other hedgehog studies (0.45 ± 0.02 mg/kg dw [5]; 0.69 ± 0.13 mg/kg dw [6]).

Cd is a heavy metal with no biological function, and exposure may occur through inhalation or ingestion. The incidence of acute toxicity is rare (e.g., inhalation of Cd fumes), but chronic toxicity is reflected in continuing accumulation of the metal from the diet in the liver and subsequent tissue dysfunction [23]. Data from small mammals (mice, rats and voles) suggest that average Cd levels in the liver should range from 0.2 to 1.5 mg/kg dw [23]. In the present study, animals without biliary hyperplasia presented an average Cd level of 0.46 ± 0.59 mg/kg dw, which is included in this interval. However, animals presenting biliary hyperplasia showed a mean value of 1.57 ± 1.85 mg/kg dw, and a maximum value of 6.07 mg/kg dw, which are above the normal limits. Therefore, Cd exposure may be a cause of or factor contributing to biliary hyperplasia in these hedgehogs. Nevertheless, these authors also mention that animals with a stricter insectivorous diet (such as moles and shrews) may accumulate higher levels of Cd (2.3–25.4 mg/kg dw) [23]. Although insects are a considerable part of their diet, hedgehogs do not have a strict insectivorous diet, as they also eat fruits, vegetables and eggs [24], so it is difficult to decide which one of these reference intervals should be used. Specifically in hedgehogs, D’Havé et al. [6], in Belgium, reported a mean value of 13.39 ± 3.09 mg/kg dw of Cd in the liver; and Rautio et al. [5], in Finland, reported 1.81 ± 0.20 mg/kg dw, which reflects the disparity in values that Cd may assume in this species, depending on several factors (including provenance). Similarly, Rautio et al. [5] reported a strong tendency for Cd to accumulate with age, with linear regression, which is in accordance with our significant difference between the group of adults and the group of cubs and juveniles.

Co (specially CoCl2) has been associated with hepatocellular injuries and -ns [16], even though the literature is scarce for this element. For human and animal populations, diet (including drinking water) is the main source of Co [25]. In hedgehogs, the only mean value of Co found in the literature was 0.40 ± 0.04 mg/kg dw [6], which is exactly the same mean value we found in hedgehogs with biliary hyperplasia (0.40 ± 0.42 mg/kg dw), but higher than the one we obtained for the whole group of hedgehogs (0.27 ± 0.28 mg/kg dw). To the authors’ knowledge, no studies have reported a tendency of Co to accumulate in older individuals or cause biliary hyperplasia, despite the statistical results found in this study.

Cr normally occurs as Cr^VI^ or Cr^III^. While Cr^VI^ is considered highly toxic and carcinogenic, Cr^III^ is an essential element for mammals in small amounts. However, there is a lack of knowledge regarding the normal values of these compounds, and regarding the adequate intake limits, even for humans [26]. Compared to other studies in hedgehogs (3.9 ± 0.2 mg/kg) [6] or shrews (3.00 ± 0.48 mg/kg) [27] from other locations, the Cr levels presented in this study are considerably lower (0.12 ± 0.11 mg/kg). The use of fertilizers with Cr can significantly increase the amount of Cr in soils and water, leading to substantial differences in distinct animal populations, depending on their habitats [27,28], which may explain these divergences.

Cu is a cofactor of multiple enzymes. Cu liver levels were 35.66 ± 19.65 mg/kg dw, and some hedgehogs’ levels surpassed 100 mg/kg dw. These liver values are high compared to other studies on insectivores that compared multiple locations, such as those on shrews (*Sorex araneus*, in which the highest mean value was 32.5 mg/kg dw) [29] and moles (*Talpa europaea*, in which the highest mean value was 18.6 mg/kg dw) [30]. Chronic exposure to large amounts of Cu may cause toxicosis in wild animals. Cu accumulates in hepatocytes, leading to hepatocellular damage; non-functional hepatic metabolism; deficient biliary and urinary excretion; and imbalances with other trace elements. Nevertheless, it seems that Cu levels need to be extremely high to be responsible for clinical toxicity. In New Zealand, wild rabbits (*Oryctolagus cuniculus*) with evident Cu toxicosis presented 319 to 997 mg/kg wet weight (ww) (reference range: 8–50 mg/kg ww) [31].

Pb is possibly one of the most well-studied heavy metal(loid)s (and Hg). However, the exact molecular mechanisms involved in its toxicity are not completely understood yet [12]. The present study revealed a mean value of 0.54 ± 0.70 mg/kg, considering all hedgehogs. Values in mammals are extremely variable, depending on the species and geographical areas. Average levels of 3.3 mg/kg dw were detected in mice and voles with unaltered birth and litter survival rates [32]. Biliary hyperplasia (and other important histopathologic alterations) was found in laboratory rats with 3.424 ± 0.090 mg/kg [33]. However, the risk of exposure for rodents is considered lower than for insectivores because of the higher metabolic rates and dietary requirements of insectivorous species [32]. In moles, females also had higher Pb concentrations than males, and authors discarded the possibility of an artefact, mentioning that lactation in female moles may affect Ca and Pb metabolism [34]. Possibly, the same could have happened with these hedgehogs, explaining the sex differences found in the present study.

Animals with biliary hyperplasia presented higher metal(loid) concentrations in the liver (except for As), and the difference was statistically significant for Cd and Co. Biliary hyperplasia induced by chronic or acute exposure to heavy metal(loid)s is not frequently described, but it has been reported in laboratory rats [33,35,36], laboratory rabbits [37], wild rabbits [31] and quails [38]. To the authors’ knowledge, specifically in hedgehogs, biliary hyperplasia was never associated with heavy metal(loid)s exposure, even though this lesion has been recently found in another pathological survey in Portugal as well [39]. Therefore, exposure to metal(loid)s could be the primary or a contributing reason for this hepatic lesion. Nevertheless, others should not be excluded, such as other chemical and toxic insults [40,41], cholelithiasis, sclerosing cholangitis and trematode infestation [42].

The obtained Hosmer and Lemeshow significance level of 0.993 indicates that the model fits the data well, meaning that the predicted probabilities are similar to the observed probabilities. However, the percentage correct of 75.7% suggests that the model can only correctly classify 75.7% of cases, without significant predictors. As mentioned, Log10Co resulted in the highest Exp(B) value of 16.392. Log10Pb resulted in the second highest Exp(B) value of 9.248. This means that the odds of the outcome variable occurring (in other words, the presence of BH) increase by a factor of 16.392 or 9.248 for every one-unit increase in Log10(Co) or Log10(Pb), respectively.

Further research is needed to clarify the molecular mechanisms induced by heavy metal(loid)s that may be responsible for histopathologic alterations, such as biliary hyperplasia. Moreover, the measurements of enzymes (such as ALT, AST or ALP) and their correlations with metal(loid) levels may also represent a possible study and opportunity to include living individuals in future biomonitoring studies. Other aspects of hedgehogs’ health and fitness should also be considered in other studies reporting hepatic lesion and metal(loid) assessments. Biochemical biomarkers (e.g., oxidative stress and lipid peroxidation markers) may also be applied to wildlife biomonitoring studies, in order to improve our knowledge of the exact mechanisms of hepatotoxicity induced by these compounds in species of interest (such as hedgehogs).

## 5. Conclusions

Hedgehogs with biliary hyperplasia presented higher metal(loid) concentrations in the liver (except for As), and the difference was statistically significant for both Cd and Co. Moreover, the mean values for all metal(loid)s were higher in adults compared to hoglets and juveniles; and no statistically significant differences were found between males and females (except for Pb).

This study suggests that heavy metal(loid)s may represent a primary cause or a factor contributing to biliary hyperplasia in hedgehogs. Notwithstanding, further research (including the use of biochemical biomarkers of oxidative stress and lipid peroxidation) is necessary to support these findings and contribute to a better understanding of the exact mechanism(s) that leads to liver and biliary-system cellular injury induced by metal(loid)s. Biomonitoring studies with sentinel species (such as hedgehogs) will contribute to a better comprehension of this hazard of One Health importance.

## Figures and Tables

**Table 1:** Summary of metal(loid)s’ concentrations in liver samples (mg/kg dw).

		n	Mean	SD	Min.	Max.
As	without BH	27	0.13	0.16	0.00	0.69
	with BH	14	0.13	0.11	0.00	0.44
	Total	41	0.13	0.14	0.00	0.69
Cd	without BH	27	0.46	0.59	0.00	1.84
	with BH	14	1.57	1.85	0.00	6.07
	Total	41	0.84	1.27	0.00	6.07
Co	without BH	27	0.19	0.13	0.05	0.46
	with BH	14	0.40	0.42	0.06	1.66
	Total	41	0.27	0.28	0.05	1.66
Cr	without BH	27	0.12	0.13	0.02	0.70
	with BH	14	0.13	0.07	0.03	0.29
	Total	41	0.12	0.11	0.02	0.70
Cu	without BH	27	33.06	21.42	12.11	102.91
	with BH	14	40.68	16.09	13.60	65.75
	Total	41	35.66	19.89	12.11	102.91
Pb	without BH	27	0.40	0.25	0.11	1.16
	with BH	14	0.80	1.12	0.09	4.46
	Total	41	0.54	0.70	0.09	4.46

BH—biliary hyperplasia.

**Table 2:** ANOVA test summary results for log-transformed (Log_10_(x)) metal(loid)s’ concentrations.

		N	Mean	SD	Min.	Max	ANOVA					
								Sum of Squares	df	Mean Square	F	Sig.
Log_10_(As)	without BH	24	−0.98	0.37	−1.72	−0.16	Between Groups	0.003	1	0.003	0.026	0.873
	with BH	13	−0.96	0.31	−1.47	−0.36	Within Groups	4.344	35	0.124		
	Total	37	−0.98	0.35	−1.72	−0.16	Total	4.348	36			
Log_10_(Cd)	without BH	26	−0.81	0.74	−1.90	0.26	Between Groups	4.137	1	4.137	8.122	0.007 **
	with BH	13	−0.11	0.65	−1.37	0.78	Within Groups	18.846	37	0.509		
	Total	39	−0.58	0.78	−1.90	0.78	Total	22.983	38			
Log_10_(Co)	without BH	27	−0.80	0.29	−1.27	−0.34	Between Groups	0.607	1	0.607	6.001	0.019 *
	with BH	14	−0.55	0.37	−1.19	0.22	Within Groups	3.944	39	0.101		
	Total	41	−0.72	0.34	−1.27	0.22	Total	4.551	40			
Log_10_(Cr)	without BH	27	−1.06	0.32	−1.71	−0.16	Between Groups	0.065	1	0.065	0.704	0.407
	with BH	14	−0.97	0.27	−1.53	−0.54	Within Groups	3.594	39	0.092		
	Total	41	−1.03	0.30	−1.71	−0.16	Total	3.658	40			
Log_10_(Cu)	without BH	27	1.46	0.21	1.08	2.01	Between Groups	0.115	1	0.115	2.733	0.106
	with BH	14	1.57	0.19	1.13	1.82	Within Groups	1.639	39	0.042		
	Total	41	1.50	0.21	1.08	2.01	Total	1.754	40			
Log_10_(Pb)	without BH	27	−0.47	0.25	−0.95	0.06	Between Groups	0.161	1	0.161	1.459	0.234
	with BH	14	−0.34	0.45	−1.03	0.65	Within Groups	4.308	39	0.110		
	Total	41	−0.42	0.33	−1.03	0.65	Total	4.470	40			

BH—biliary hyperplasia. *: *p* < 0.05; **: *p* < 0.01.

## Data Availability

There is no data available from this study.

## Appendix A. Binary Logistic Regression Model Results

**Table A1:** Model classification table ^a^.

Observed			Predicted		
			BH		Percentage Correct
			No	Yes	
Step 1	BH	No	21	3	87.5
		Yes	6	7	53.8
	Overall Percentage				75.7

a. The cut value is 0.500.

**Table A2:** Hosmer and Lemeshow test results.

Step	Chi-Square	df	Sig.
1	1.077	7	0.993

**Table A3:** Variables in the equations (Exp(B) values of Pb and Co underlined).

		B	S.E.	Wald	df	Sig.	Exp(B)	95% C.I.for EXP(B)	
								Lower	Upper
Step 1	sex(1)	−2.700	1.402	3.706	1	0.054	0.067	0.004	1.050
	age			0.019	2	0.990			
	age(1)	−0.212	1.708	0.015	1	0.901	0.809	0.028	23.016
	age(2)	−0.174	1.458	0.014	1	0.905	0.840	0.048	14.652
	Log10As	−1.057	1.665	0.403	1	0.525	0.347	0.013	9.082
	Log10Cd	0.337	1.189	0.080	1	0.777	1.401	0.136	14.416
	Log10Co	2.797	1.930	2.100	1	0.147	16.392	0.373	720.297
	Log10Cr	0.267	2.011	0.018	1	0.894	1.306	0.025	67.179
	Log10Cu	1.905	2.557	0.555	1	0.456	6.722	0.045	1009.238
	Log10Pb	2.224	1.685	1.742	1	0.187	9.248	0.340	251.525
	Constant	−0.243	4.242	0.003	1	0.954	0.784		
